# Novel compound heterozygous synonymous and missense variants in the *MYO7A* gene identified by next‐generation sequencing in a Chinese family with nonsyndromic hearing loss

**DOI:** 10.1002/jcla.24708

**Published:** 2022-09-26

**Authors:** Yanbao Xiang, Chenyang Xu, Yunzhi Xu, Lili Zhou, Shaohua Tang, Xueqin Xu

**Affiliations:** ^1^ Department of Genetics, Key Laboratory of Birth Defects of Wenzhou Wenzhou Central Hospital Wenzhou China; ^2^ Department of Clinical Laboratory Medicine, Key Laboratory of Precision Medicine of Wenzhou Wenzhou Central Hospital Wenzhou China

**Keywords:** DFNB2, minigene splicing assay, *MYO7A* gene, nonsyndromic hearing loss, synonymous variant

## Abstract

**Background:**

Variants in the *MYO7A* gene are increasingly identified among patients suffering from Usher syndrome type 1B (USH1B). However, such mutations are less commonly detected among patients suffering from nonsyndromic hearing loss (NSHL), including autosomal recessive deafness (DFNB2) and autosomal dominant deafness (DFNA11). This research attempts to clarify the genetic base of DFNB2 in a Chinese family and determine the pathogenicity of the identified mutations.

**Method:**

Targeted next‐generation sequencing (TGS) of 127 known deafness genes was performed for the 14‐year‐old proband. Then, Sanger sequencing was performed on the available family members. A minigene splicing assay was performed to verify the impact of the novel *MYO7A* synonymous variant. After performing targeted next‐generation sequencing (TGS) of 127 existing hearing loss‐related genes in a 14‐year‐old proband, Sanger sequencing was carried out on the available family members. Then, to confirm the influence of the novel *MYO7A* synonymous variants, a minigene splicing assay was performed.

**Results:**

Two heteroallelic mutants of *MYO7A* (NM_000260.3) were identified: a maternally inherited synonymous variant c.2904G > A (p.Glu968=) in exon 23 and a paternally inherited missense variant c.5994G > T (p.Trp1998Cys) in exon 44. The in vitro minigene expression indicated that c.2904G > A may result in skipping of exon 23 resulting in a truncated protein.

**Conclusions:**

We reported a novel missense (c.5994G > T) and identified, for the first time, a novel pathogenic synonymous (c.2904G > A) variant within *MYO7A* in a patient with DFNB2. These findings enrich our understanding of the *MYO7A* variant spectrum of DFNB2 and can contribute to accurate genetic counseling and diagnosis of NSHL patients.

## Graphical abstract

This study describes a novel c.5994G > T (p.Trp1998Cys) missense variant and reported, for the first time, a synonymous coding base change c.2904G > A (p.Glu968=) variant within *MYO7A* in a patient with nonsyndromic hearing loss (DFNB2 phenotype). The in vitro minigene expression indicated that p.Glu968= could cause skipping over exon 23, which was predicted to result in a truncated protein. Further, the 3D structures of the homomeric wild‐type and mutated *MYO7A* showed that the substitution of p.Trp1998Cys was likely to disrupt intramolecular and intermolecular interactions between Trp1998 and its neighboring residues. These findings enrich our understanding of the *MYO7A* variant spectrum of DFNB2 and can contribute to accurate genetic counseling and diagnosis of nonsyndromic hearing loss patients.

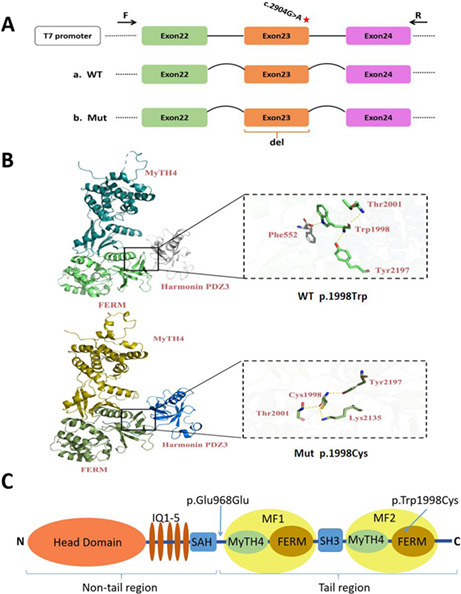

## 1 INTRODUCTION

Hearing loss (HL) is a disorder of human perception that exhibits extreme genetic and clinical heterogeneity. Data from the World Health Organization (WHO) indicate that nearly 5% of the world's population has HL.^1^ HL has both genetic and environmental contributors; genetic cases of HL account for more than 50% of HL cases.^2^, ^3^ Further, approximately 30% of hereditary HL is syndromic and presents with other complications, while the other 70% is nonsyndromic.^4^ Research has found more than 110 genes and 150 loci to be related to HL (https://hereditaryhearingloss.org/). Among Chinese patients with nonsyndromic hearing loss (NSHL), *GJB2*, *SLC26A4*, and mtDNA *12SrRNA* are the most frequently identified genes, representing 40%–50% of cases.^5^, ^6^ The remainder of cases result from other rare mutations of HL genes or other unidentified etiologies.^7^, ^8^

The human *MYO7A* gene is located on chromosome 11q13.5 and encodes the actin‐binding dynamic protein myosin VIIa, which belongs to the unconventional myosin family.^9^ It is composed of 2215 amino acids and influences retinal pigment epithelium, retinal photoreceptor cells, and cochlea and vestibular neuroepithelia.^9^, ^10^ Thus, *MYO7A* pathogenic variants can cause both syndromic and NSHL, including autosomal dominant nonsyndromic hearing loss (ADNSHL, DFNA11), autosomal recessive nonsyndromic hearing loss (ARNSHL, DFNB2), and syndromic deaf‐blindness (Usher Type 1B, USH1B).^11^, ^12^, ^13^ Studies have identified an interaction between the myosin VIIa protein, the SANS (encoded by USH1G gene) protein, and the harmonin (encoded by USH1C gene); this triplet in hair cell stereocilium transports the protein complex to the tip which, in turn, regulates mechanoelectrical transduction (MET).^14^, ^15^ The *MYO7A* protein contains a domain that provides its motor and cargo binding functions.^16^, ^17^ The intact head domain includes an ATP binding site and an actin‐binding site in the N‐terminal area and the neck region, which is composed of five isoleucine‐glutamine (IQ) motifs to which calmodulin binds. They are then followed by a steady single α‐helix (SAH) and a pair of myosin tail homology 4 (MyTH4) domains and integrant 4.1‐ezrinradixin‐moesin (FERM) domains, which are screened off by an SH3 domain in the tail domain of the C‐terminal region.^16^, ^17^ It has been demonstrated that the tail domain regulates dimerization and binding with different cargo and proteins; this plays an important role in *MYO7A* protein movement.^16^

According to the Human Gene Mutation database (HGMD; http://www.hgmd.cf.ac.uk/ac/all.php; professional version dated 2021.04), 427 deleterious variants on the *MYO7A* gene have been found to be associated with HL to date. In contrast, <10% of these variants are responsible for the clinical phenotype of NSHL, and none of these variants in NSHL patients are synonymous. As synonymous variants do not exhibit altered amino acid sequences and most are considered benign or possibly benign, they are often filtered in bioinformatic analyses in accordance with the American College of Medical Genetics and Genomics/Association for Molecular Pathology (ACMG/AMP) recommendations and ClinGen Hearing Loss Expert Panel specifications.^18^, ^19^

**FIGURE 1:**
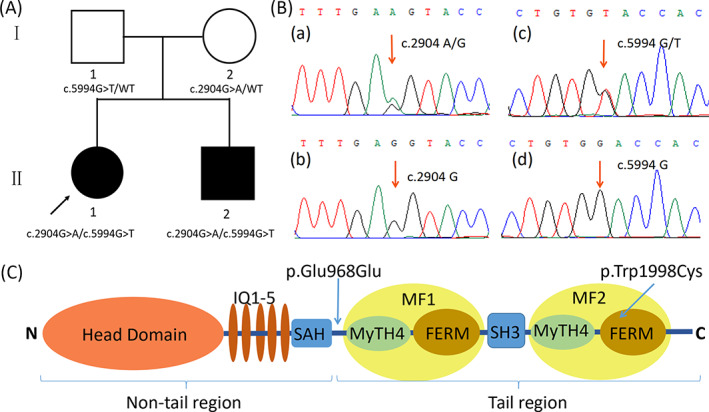
(A) Pedigree and genotype data of all family members; (B) Electropherogram presenting the *MYO7A* wild‐type sequences and heterozygous mutations identified in the DFNB2 family; (C) Domains with mutations in the *MYO7A* protein

This study describes the genetic, molecular, and clinical features of a Chinese Han family with ARNSHL. In a previous investigation, the proband of the family was not found to harbor any of the common mutant deafness genes, including *GJB2* (c.35delG, c.176_191del16, c.235delC, c.299‐300delAT), *SLC26A4* (c.919‐2A > G, c.1174A > T, c.1226G > A, c.1229C > T, c.1707 + 5G > A, c.1975G > C, c.2027 T > A, c.2168A > G), and *MT‐RNR1* (m.1494C > T, m.1555A > G), and *GJB3* (c.538C > T). Thus, we carried out targeted next‐generation sequencing (TNGS) to identify whether the proband harbored any rare pathogenic variants. The results revealed that the proband harbored a compound heterozygous maternally inherited variant, c.2904G > A (p.Glu968=), and a paternally inherited variant, c.5994G > T (p.Trp1998Cys, NM_000260.3), in the *MYO7A* gene (NM_000260.3). c.5994G > T is a missense mutation at exon 44 of the *MYO7A* gene, whereas the c.2904G > A variant is a synonymous variant at the last base of exon 23 (Figure 1). Both variants are novel and have not been reported in HL patients before. To determine whether the two variants were pathogenic sites for the proband, an *MYO7A* gene c.2904G > A and c.5994G > T mutation pathogenicity study was performed.

## 2 MATERIALS AND METHODS

### 2.1 Patients and clinical information

A nonconsanguineous Chinese Han family containing two siblings, a daughter (14‐year old) and a son (two‐year old), both suffering from congenital bilateral HL, took part in this study. The proband (the elder sister) and her family members underwent hereditary hearing impairment mutation screening at the Department of Genetics, Wenzhou Central Hospital (Figure 1A). The proband underwent comprehensive clinical examinations. High‐resolution computed tomography (HRCT) of the proband's temporal bones was performed to exclude vestibular aqueduct enlargement or other malformations of the inner ear. Tandem gait and Romberg testing were performed to assess her vestibular function. An ophthalmologic evaluation, including dilated fundus ophthalmoscopy and best‐corrected visual acuity measurement, was also performed. To examine the proband's degree of HL, pure‐tone audiometry (PTA) was performed, and the auditory brainstem response (ABR) was evaluated. The classification of HL severity was performed as follows: complete (95 dB or greater), profound (80 to <95 dB), severe (65 to <80 dB), moderately severe (50 to <65 dB), moderate (35 to <50 dB), and mild (20 to <35 dB). This research was approved and supported by the proband, her family, and the Ethics Committee of Wenzhou Central Hospital.

### 2.2 TNGS, data analysis, and co‐segregation

Blood samples were obtained from each parent and both affected patients. Genomic DNA was extracted from the whole blood using a Qiagen DNA Blood Midi/Mini Kit (Qiagen). For TNGS, a customized human array was built with Roche NimbleGen to target the exons and 10 bp flanking intron sequences of 127 genes that are implicated in deafness (Table S1). DNA sequencing was conducted using the HiSeq2000 platform (Illumina). After quality control, the Burrows–Wheeler Aligner (BWA) was used to further align the clean data in accordance with the human genome reference sequence hg19 (GRCH37). SAMtools and GATK were used to annotate the BAM files. Potential pathogenic variants were identified, based on a frequency lower than 0.005, using NCBI dbSNP (http://www.ncbi.nlm.nih.gov/snp), the Genome Aggregation database (http://gnomAD.broadinstitute.org/), and the 1000 Genomes Project database (http://browser.1000genomes.org). Using the mutation databases HGMD and Clinvar (http://www.ncbi.nlm.nih.gov/clinvar), previously documented HL pathogenic mutations were identified. Then, synonymous variations in coding regions, untranslated regions, and introns (except for splice mutations that could create ectopic sites or disrupt ordinary splice sites) were screened out. Prediction of the pathogenicity of missense variants was performed using Polyphen‐2, SIFT, and Mutation Taster. The bioinformatics splicing tool MaxEntScan algorithms was used for prediction of the functional effect of splicing site variants (if the value ∆MaxEnt = MaxEntvar‐MaxEntref <0, a variant sequence was denoted as a potential deleterious site that may disrupt of potential acceptor or donor sites splicing).^20^, ^21^ Conservation analyses were carried out using the NCBI database by aligning the amino acid sequences of MYO7A proteins among multiple diverse species. Given the proband's family history, we suspected a recessive inheritance model; thus, compound heterozygous or homozygous mutations were selected. Sanger sequencing of the proband's family was performed to determine co‐segregation of the disease phenotype and candidate mutations. The primers included the following: *MYO7A*‐2904‐F/R: CCTCTGAGTGGTCCAGAGGT/TCTCTGCTCCCCACTGTTCA; *MYO7A*‐5994‐F/R: GACGTGAGC ACTCCTCTGTG/GCCTGAACAGGTAGGG. The variants identified were eventually categorized with reference to ACMG/AMP recommendations and ClinGen Hearing Loss Expert Panel specifications.^18^, ^19^

In addition, to exclude potential causative copy number variants (CNVs) that may have caused the HL phenotype in the proband, chromosomal microarray analysis (CMA) was performed using Affymetrix CytoScan 750 k Array, according to the manufacturer's instructions.

### 2.3 Molecular modeling

In order to predict the influence of p.Trp1998Cys amino acid substitution on the *MYO7A* protein structure, a molecular modeling method was employed. We constructed the 3D structures of the homomeric wild‐type and mutated *MYO7A* MyTH4‐FERM tandem domain (MF2) using the Swiss‐Model Server (http://swissmodel.expasy.org), and the structures were illustrated using the PyMol Molecular Graphic system. The resolved structure of Myosin VIIa encoded by the *MYO7A* gene was used as a template (Protein Data Bank No.5mv9, a complex of harmonin‐a PDZ3 domain and human Myosin VIIa C‐terminal MyTH4‐FERM domain)

### 2.4 Minigene construction and expression

The synonymous variant c.2904G > A (p.Glu968=) situated at the last base of exon 23, which is a highly conserved base that is part of the 5′ splice site. This variant was selected based on bioinformatic prediction. To analyze the influence of the variant on splicing, DNA fragment exons 22–24, including exon 22, intron 22, exon 23, intron 23, and exon 24 were amplified using the following primers: *MYO7A*‐exon22‐F: gagacccaagctggctagcgccaccATGTATCTGTGGCGCCTCGAGGCTG and *MYO7A*‐exon24‐R: cttggtaccgagctcggatccCAGCTGGTCACCCTCGTCGTCATG (lowercase letters in primer F and R indicate the addition of nucleotides to the primers to depict the NheI and BamHI restriction sites, respectively). The PCR product was distilled and embedded in the pCDNA3.1 vector that was digested with NheI and BamHI through recombination. The designated clones were then sequenced by Sanger sequencing to verify that the mutant‐type (pcDNA3.1‐*MYO7A*‐Mut) and wild‐type clones (pcDNA3.1‐*MYO7A*‐Wt) were successfully obtained.

Using standard procedures, HEK293T (human embryonic kidney 293 T) cells were cultured in Dulbecco's minimum essential medium (DMEM) comprising 10% fetal bovine serum (Invitrogen, Carlsbad, USA) at 37°C in a 5% CO_2_ atmosphere. Then, Lipofectamine 2000 (Invitrogen) was used to transiently transfect of 4 μg wild‐type and mutant minigene constructs into HEK293T cells. 48 h after transfection, TRIzol reagent (Invitrogen) was employed to extract total RNA from the HEK293T cells. Further, reverse transcription of 2 μg of total RNA was performed using the reverse transcription system Takara. For RNA retrotranscription, 200 ng of cDNA from the wild‐type and mutate minigene constructs, respectively, was PCR amplified using primers Det‐Mini‐F:CTATAGGGAGACCCAAGCT and Det‐Mini‐R:CAACTAGAAGGCACAGTCG. 2% agarose gel electrophoresis was used to confirm and sequence the PCR product.

## 3 RESULTS

### 3.1 Clinical features

The affected individuals (II1 andII2) were born to a healthy nonconsanguineous Chinese family with congenital bilateral HL. PTA and ABR revealed that the proband had complete sensorineural HL with average thresholds >100 dB in both the left and right ear. No HL features were evident in the parents. An HRCT scan of the temporal bone did not indicate any inner ear malformities. The middle ear structures and ossicles also appeared normal. A clinical examination, including vestibular function evaluation and visual examination (Figures S1–S2), of the proband appeared to be normal, with no deformities suggestive of syndromic HL. Thus, the results of the clinical examination and audiological evaluation of the proband were in accordance with the clinical diagnosis of NSHL.

### 3.2 TNGS results and co‐segregation

TNGS was performed in the proband. A mean depth of 225.17 for the covered exons was achieved, and 99.95% of the bases were covered by at least 30 sequencing reads. In view of the filter criteria and recessive genetic model, two novel compound heterozygous mutants, c.2904G > A (p.Glu968=) and c.5994G > T, in the *MYO7A* gene of the proband, were suspected to be potentially related to the disease. No other pathogenic variants in HL genes were identified. No potential pathogenic CNVs were detected by chromosomal microarray analysis (Figure S2). The missense variant c.5994G > T (p.Trp1998Cys) was absent from the dbSNP, 1000 Genomes Project, and gnomAD databases. The synonymous variant c.2904G > A was not identified in the 1000 Genomes Project database but was registered in dbSNP (rs111033233) with a relatively low frequency in the global population of gnomAD (0.0000041). Sanger sequencing of *MYO7A* showed that both the proband and her brother had compound heterozygous variations of c.2904G > A and c.5994G > T, whereas the healthy parents carried the heterozygous c.5994G > T variant and the heterozygous c.2904G > A variant, respectively (Figure 1A/B). These results suggest that the compound heterozygous variants (c.2904G > A and c.5994G > T) are co‐segregated with the clinical phenotype and could lead to deafness in the next generation.

### 3.3 Bioinformation analysis and structure modeling

**FIGURE 2:**
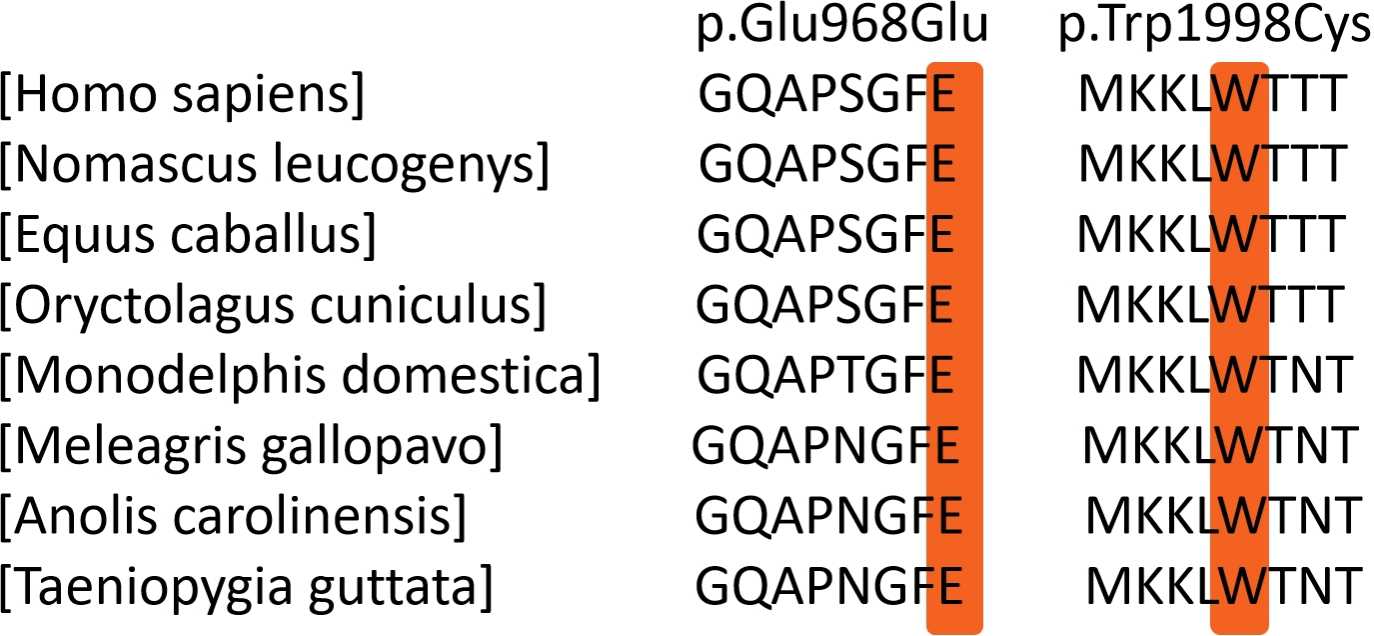
Alignment of the *MYO7A* amino acid sequences in diverse species. The amino acid variants are highly conserved residues

Conservation analysis results revealed that the aromatic amino acid tryptophan at site 1998 (p.Trp1998) is highly conserved across species (Figure 2), and its replacement by the sulfur amino acid cysteine was predicted to be deleterious using Polyphen‐2 (0.997, possibly destructive), SIFT (0.000, destructive), and Mutation Taster (disease‐causing) in silico prediction software. For the c.2904G > A variant, substitution occurred at the last base of exon 23 and did not change the amino acid sequence of site 968 (p.Glu968=); yet, it was suggested to affect splicing with disruption of the exon 23 donor site, according to the MaxEntScan algorithm (MaxEntvariant value −2.09; MaxEntreference value 6.43; ∆MaxEnt = −8.52 < 0).

**FIGURE 3:**
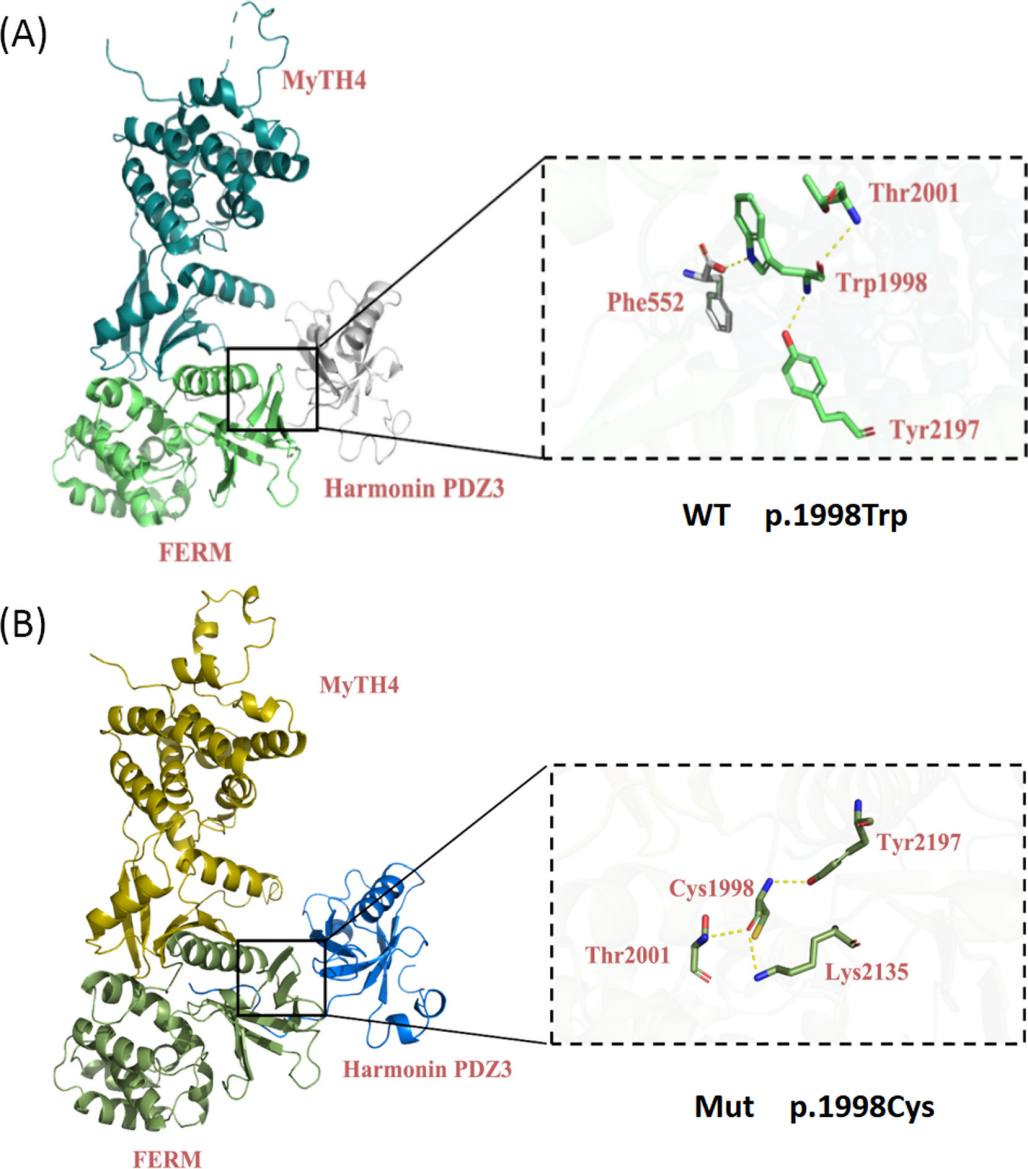
Functional and structural influences of the *MYO7A* c.5994G > T (p.Trp1998Cys) missense mutation, as predicted by molecular modeling

The crystal structure of MYO7A MyTH4‐FERM (Protein Data Bank No.5mv9) was used as a template for modeling the second MyTH4‐FERM tandem domain. The alternation of p.Trp1998Cys may influence intramolecular interactions between Trp1998 and its neighboring residues: Trp1998 forms hydrogen bonds with Thr2001, Tyr2197, and the harmonin (*USH1C*) PDZ3 domain Phe552. Cys1998 significantly changes the hydrogen bonds. The interaction with Thr2001 and Tyr2197 remains, but with the harmonin PDZ3 domain Phe552, the hydrogen bond disappears. In addition, a new hydrogen bond appears between Cys1998 and Lys2135 (Figure 3).

### 3.4 Splicing study of *MYO7A* c.2904G > A by minigene assay

**FIGURE 4:**
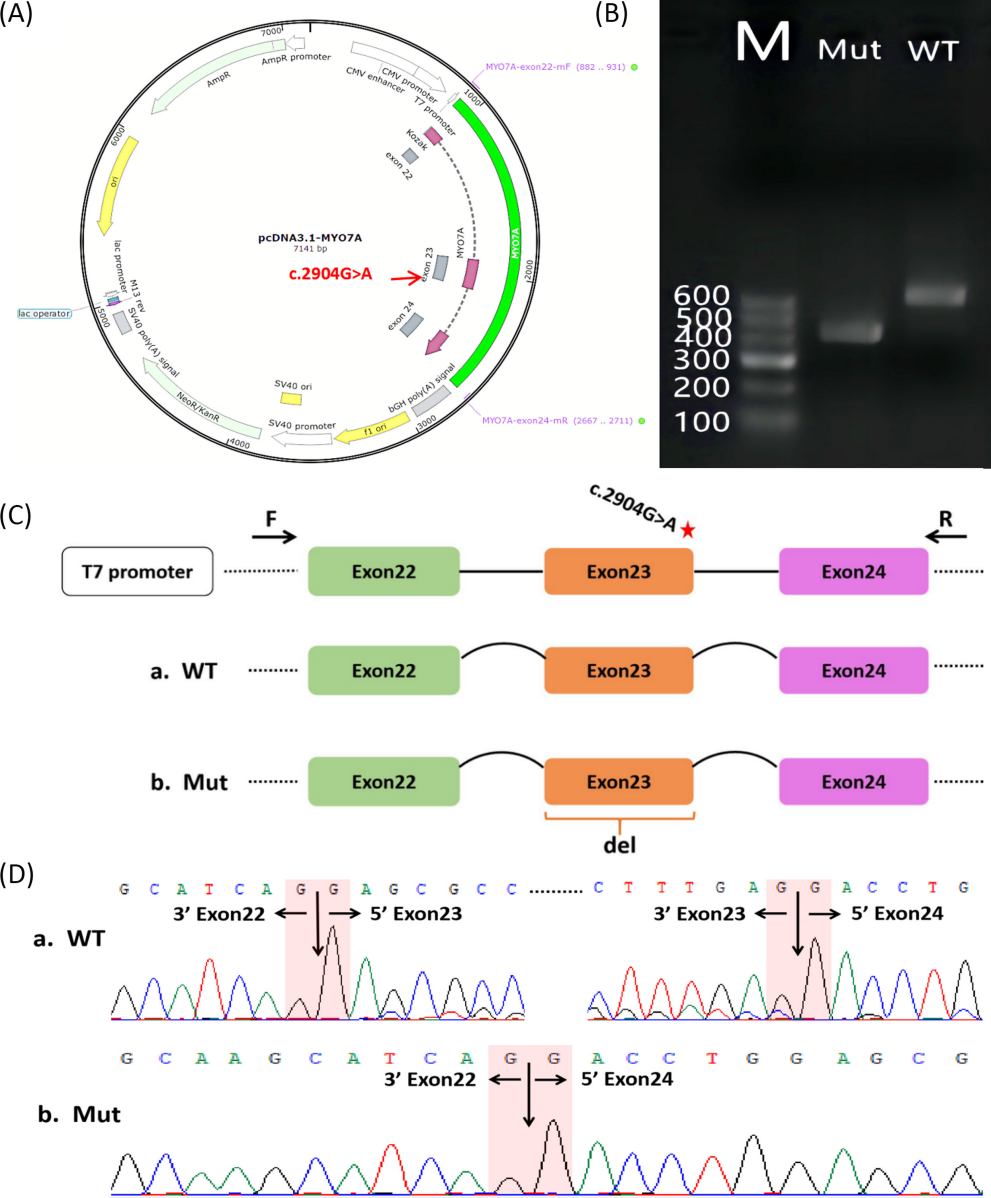
Splicing changes confirmed with minigene assay. (A) Construction of the pcDNA3.1‐*MYO7A*‐WT/Mut vector containing exon 22 to exon 24 sequences from wild‐type or mutant types (c.2904G > A) of the *MYO7A* gene; (B) Agarose gel (2%) electrophoresis of RT‐PCR products acquired from RNA of HEK 293 T cells transfected with the wild‐type or mutant‐type minigene vector; Lanes WT and Mut refer to different transfections of the wild‐type (c.2904G) or mutant‐type (c.2904A) constructs; (C) Schematic diagram of minigene construction and schematic diagram of Sanger sequencing of RT‐PCR products; (D) Sequencing traces of the normally spliced 623 bp fragment (wild‐type) and the 413 bp fragment (mutant‐type) skipping exon 23

Figure 4A illustrates the minigene structure. After cell transfection of pcDNA3.1‐*MYO7A*‐Wt, RT‐PCR of total RNA was performed, resulting in a 623 bp band consistent with the correct splicing of mRNA, and a distinct 413 bp band in cells transfected with pcDNA3.1‐*MYO7A*‐Mut (Figure 4B). After purification and sequencing of the RT‐PCR products, it appeared that the *MYO7A* synonymous mutation (c.2904G > A) resulted in the complete absence of exon 23 of the *MYO7A* gene during mRNA splicing (Figure 4C/D).

## 4 DISCUSSION

The majority of synonymous variants are considered likely benign and are often disregarded in genetic analyses as they do not alter amino acid sequences.^15^ However, synonymous variants involving disease‐causing genes have been increasingly reported in over 50 human diseases. Thus, identification of the pathogenicity of synonymous mutations could enhance gene diagnosis.^22^ The possible mechanisms of pathogenic synonymous variants have been clarified using various approaches and include changes to RNA splicing (creating a cryptic new donor/acceptor splice site resulting in aberrant splicing; loss of a classical donor/acceptor splice site resulting an exon skipping, etc.); alterations to the secondary structure of mRNA, which affects mRNA stability; regulation of the translation rate, which influences protein expression or enzymatic activity.^23^, ^24^, ^25^, ^26^ Although various computational predictions, such as Human Splicing Finder (HSF), MaxEntScan, and Splice AI, can be used to predict pathogenic synonymous variants, these predictions can be unreliable. Consequently, in vivo and (or) in vitro functional assays, such as the minigene splicing assay and protein expression experiment, must be performed to verify these predictions.^24^, ^25^ This may contribute to the exploration of pathomechanism of pathogenic synonymous variants.

At present, we identified a novel c.2904G > A (p.Glu968=) synonymous mutation and a new missense mutation in a Chinese family with NSHL using targeted 127 HL gene and next‐generation sequencing; this was then validated by Sanger sequencing. The c.2904G > A (p.Glu968=) synonymous mutation is at the last base of exon 23, which is a highly conserved base that is part of the 5′ splice region. As it involves the characteristic consensus sequence of the splicing donor site, AG/GUAAGU, mutation at this position may change the donor splice site of intron 24. The MaxEntScan algorithm also predicted that this mutation is likely to influence the splicing process by the loss of a donor splice site. To further elucidate the pathogenicity of this synonymous mutation, RT‐PCR splicing validation was performed through the construction of a minigene vector. The RT‐PCR findings indicated that the c.2904G > A variation led to abnormal splicing and the complete skipping of exon 23, thus producing incomplete myosin VIIa which is missing part of the MyTH4‐FERM 1 (MF1) subdomain (Figure 4C/D). This may significantly affect the folding of the MF1 subdomain and probably interferes with the mutual effect between the SANS protein and myosin VIIa.^15^, ^27^ Precise cooperation between the myosin VIIa, SANS and harmonin proteins is essential as they are key factors in the structure of stereocilia, and failure of this cooperation can lead to defective stereocilia and HL.^15^, ^28^

*MYO7A* c.5994G > T (p.Trp1998Cys) is located in the second MyTH4‐FERM (MF2) subdomain. It is known to play a vital role in the interaction with the harmonin (*USH1C*) PDZ3 domain.^15^, ^27^, ^28^ Mutations occurring in MF2 are reported to perturb harmonin binding, which is likely the molecular basis underlying disruption of the tripartite complex assembly and mechanotransduction function.^16^, ^29^ As shown in the crystal structure model, the substitution of p.Trp1998Cys may hinder intramolecular and intermolecular relationships between Trp1998 and its neighboring residues: Trp1998 forms hydrogen bonds with Thr2001, Tyr2197, and the harmonin (*USH1C*) PDZ3 domain Phe552 (Figure 3A). For Cys1998, the interaction with Thr2001 and Tyr2197 remains, but with harmonin PDZ3 Phe552, the hydrogen bond is absent. In addition, a new hydrogen bond occurs between Cys1998 and Lys2135 (Figure 3B). Therefore, it seems that the protein subdomain interaction between myosin VIIa MF2 and harmonin PDZ3 is influenced, and intramolecular interactions among the MF2 subdomain are reinforced. These changes may cause nonfunctional tripartite complexes in the stereocilium, resulting in poor MET and even HL.

Based on the ACMG/AMP recommendations and Expert Panel specifications for interpreting genetic HL variants, a pathogenicity analysis was performed on these two mutations. For c.2904G > A: (a) an in vitro validation experiment involving the construction of a minigene vector indicated that the c.2904G > A synonymous variant caused complete skipping of exon 23 in *MYO7A*, which subsequently led to gene function impairment; this confirms the pathogenicity of the variant (PS3); (b) the c.2904G > A mutation is not present in the 1000 Genomes Project database and has a low frequency in the global population of the gnomAD database (0.0000041) (moderate pathogenic evidence PM2); (c) both the proband and her younger affected brother carry the *MYO7A* c.2904G > A and c.5994G > T compound heterozygous mutations, while their parents, free from hearing disorder, only carry one mutant allele in *MYO7A* (the father carries the *MYO7A* c.5994G > T variant while the mother harbors the c.2904G > A variant), suggesting that the phenotype of HL was co‐segregated with the *MYO7A* genotype in members of the family (supporting pathogenic evidence, PP1). Overall, the research suggests that the c.2904G > A variation is “PS3 + PM2 + PP1,” and thus, it is considered a pathogenic variation. For c.5994G > T: (a) the novel missense is not found in the dbSNP, 1000 Genomes Project, and gnomAD databases (moderate pathogenic evidence PM2); (b) the DFNB2 HL phenotype is a recessive disease and has been found to be associated with a likely pathogenic variation in c.2904G > A; thus, there is moderate pathogenic evidence (PM3) for the second allele, c.5994G > T, in the proband and her sibling; (c) the phenotype of HL was co‐segregated with the *MYO7A* genotype in the family members (supporting pathogenic evidence, PP1); (d) c.5994G > T is conserved in several vertebrates and may lead to disease according to the prediction results of Polyphen‐2, SIFT, and Mutation Taster. The crystal structure analysis of this mutation also exhibited altered intra‐ or intermolecular interactions, as compared to wild‐type myosin VIIa (supportive pathogenic evidence, pp3). Thus, c.5994G > T was also identified as “likely pathogenic” based on the above “PM2 + PM3 + PP1 + PP3” evidence.

To date, hundreds of *MYO7A* variations have been detected in people suffering from Usher syndrome, and about 30 of them have been clearly characterized in 25 DFNB2 families. As there is no current treatment for retinitis pigmentosa, patients with the Usher syndrome are supposed to have worse survival quality than patients with DFNB2 phenotype. Thus, the exploration of the *MYO7A* genotype–phenotype correlation is of great significance. The recent study of DFNA11 families with *MYO7A* heterozygous mutations showed that individuals carrying myosin VIIa protein tail variants had more severe audiological phenotypes than patients with mutations in the motor domain. This suggests that audiological differences among ADNSHL patients correspond to specific domains.^29^ However, the type of *MYO7A* variant does not seem to change the onset, severity, or course of the visual disease in Usher syndrome patients, suggesting no correlation between the *MYO7A* genotype and phenotype in Usher patients.^30^ Further, compared with Usher syndrome, there are few differences in the spatial distribution, site, or type of variants that cause DFNB2, according to a report by Kabahuma et al.^12^ In this research, we hypothesized that the mutations identified in the previously reported 25 DFNB2 families (Table 1) could be divided into tail region (including MF1 and MF2 subdomains) mutations and non‐tail region (including motor, IQ1‐5, and SAH subdomains) mutations (Figure 1C). We were surprised to find that biallelic mutations in the 22/25 families were derived from or have affected the identical myosin VIIa region (non‐tail + non‐tail or tail + tail), indicating that mutations occurring in identical tail or non‐tail regions may be more likely to result in the DFNB2 phenotype in comparison with mutations detected in the alien region (Tables 1 and 2). The findings of this research are in line with our prior research which found that both c.2904G > A and c.5994G > T affect identical tail region function, suggesting a tail + tail pattern. The hypothesis should be further verified by more data from DFNB2 families.

**TABLE 1:** Reported *MYO7A* mutations and their mutation regions in 25 DFNB2 families

No.	Population group	Family ID	Allele 1 (Nucleotide/protein)	Allele 2 (Nucleotide/protein)	Mutation region	Reference
1	Chinese	F01	c.731G > C/p.R244P	c.731G > C/p.R244P	Non‐tail + non‐tail	31
2	Chinese	F05	c.133‐2A > G/Splice region	c.3597insT/p.V1199insT	Non‐tail + tail	31
3	Pakistani	PKDF034	c.5142_5144del/p.E1716del	c.5142_5144del/p.E1716del	Tail + tail	32
4	Iraqi	F1	c.1592_1593insAG/p.C652Gfs*11	c.1592_1593insAG/p.C652Gfs*11	Non‐tail + non‐tail	33
5	Palestinians	F2	c.5660C > T/p.P1887L	c.5660C > T/p.P1887L	Tail + tail	33
6	Jewish	D75	c.620A > G/p.Asn207Ser	c.620A > G/p.Asn207Ser	Non‐tail + non‐tail	33
7	Jewish	D79	c.29 T > C/p.Val10Ala	c.1969C > T/p.Arg657Trp	Non‐tail + non‐tail	34
8	Palestinians	QS004	c.4153‐2A > G/Splice region	c.4153‐2A > G/Splice region	Tail + tail	34
9	Palestinians	QS025	c.6211C > T/p.Gln2071*	c.6211C > T/p.Gln2071*	Tail + tail	34
10	Moroccan	SF01	c.6025delG/p.Ala2009Profs*32	c.6229 T > A//p.Trp2077Arg	Tail + tail	35
11	Moroccan	SF42‐1	c.3500 T > A/p.Leu1167His	c.4487C > A/p.Thr1496Lys	Tail + tail	35
12	Moroccan	SF42‐2	c.3500 T > A/p.Leu1167His	c.5617C > T/p.Arg1873Trp	Tail + tail	35
13	Iranian	F13	c.6487G > A/p.G2163S	c.6487G > A/p.G2163S	Tail + tail	36
14	Iranian	F32	c.448 C > T/p.Arg150*	c.448 C > T/p.Arg150X	Non‐tail + non‐tail	36
15	Chinese	G2	c.2183 T > C/p.L728P	c.2187 + 2_ + 8delTGAGCAC/Splice region	Non‐tail + non‐tail	37
16	Pakistani	F4	c.470G > A/p.S157Asn	c.470G > A/p.S157Asn	Non‐tail + non‐tail	38
17	Pakistani	F5	c.3502 C > T/p.R1168W	c.3502 C > T/p.R1168W	Tail + tail	38
18	South Africans	TS065/TS100	c.5339A > C/p.Tyr1780Ser	c.5339A > C/p.Tyr1780Ser	Tail + tail	12
19	South Africans	BS044	c.1849 T > C/p.Ser617Pro	c.1849 T > C/p.Ser617Pro	Non‐tail + non‐tail	12
20	South Africans	TS076	c.5339A > C/p.Tyr1780Ser	c.6375delC/p.Pro2126Leufs*5	Tail + tail	12
21	South Africans	TS074	c.986G > A/p.Gly329Asp	c.5339A > C/p.Tyr1780Ser	Non‐tail + tail	12
22	South Africans	TS093	c.5339A > C/p.Tyr1780Ser	c.5339A > C/p.Tyr1780Ser	Tail + Tail	12
23	South Africans	TS040	c.1554 + 7C > T/p.Tyr1780Ser	c.6375delC/p.Pro2126Leufs*5	Non‐tail + tail	12
24	South Africans	TS036	c.1118G > A/p.Arg373His	c.1142C > T/p.Thr381Met	Non‐tail + non‐tail	12
25	South Africans	TS070	c.247C > A/p.Arg83Cys	c.247C > A/p.Arg83Cys	Non‐tail + non‐tail	12

*Note*: Non‐tail mutation region: *MYO7A* mutations in Motor, IQ1‐5 and SAH subdomains; Tail mutation region: *MYO7A* mutations in MF1 and MF2 subdomains.

* stands for frameshift mutation or stop codon mutation.

**TABLE 2:** Combinatorial patterns of biallelic mutation regions and their ratios in 25 reported DFNB2 families

Biallelic mutation regions		Number of families	Total	Ratio
Identical	Non‐tail + non‐tail	10	22	88.0% (22/25)
	Tail + tail	12		
Non‐identical	Non‐tail + tail	3	3	12.0% (3/25)

*Note*: This suggests that biallelic mutations occurring in identical tail or non‐tail regions may be more likely to result in the DFNB2 phenotype in comparison with mutations detected in the alien region (88.0% vs. 12%). Non‐tail mutation: *MYO7A* mutations in Motor, IQ1‐5 and SAH subdomains; Tail mutation region: *MYO7A* mutations in MF1 and MF2 subdomain.

Although the clinical data to date indicate the NSHL phenotype in this family, the diagnosis of Usher syndrome cannot be totally excluded since *MYO7A*‐related Usher syndrome usually presents with late‐onset retinal pigmented epithelium. The clinical features of Usher syndrome vary among and between families, especially with respect to the age of onset of eye disorder.^30^, ^39^ Further, one report suggested that DFNB2 and Usher syndrome patients may share the same mutations in *MYO7A*‐mutated families.^39^ Besides, several identical mutations can cause both NSHL and Usher syndrome in different families.^12^, ^35^, ^40^ Thus, eye disorder should be followed up in this family.

In summary, this study describes a novel c.5994G > T missense variant and a synonymous coding base change c.2904G > A variant involving the splicing site in a DFNB2 patient. Minigene splicing assays are crucial in assessing the pathogenicity of synonymous mutations and help us to understand the potential involvement of these mutations in diseases. The findings of this research also broaden our knowledge about the *MYO7A* variant spectrum in DFNB2 and will assist in the provision of genetic counseling for genetic disorders related to the *MYO7A* gene.

## AUTHOR CONTRIBUTIONS

Y.B. Xiang and X.Q. Xu were in charge of the theme of the study. Y.B. Xiang, L.L. Zhou, and Y.Z. Xu were responsible for the experiments and data collection. S.H. Tang and X.Q. Xu are genetic counselors for HL patients. Experimental design and manuscript drafting were carried out by Y.B. Xiang and C.Y. Xu. All authors reviewed and submitted the final draft.

## CONFLICT OF INTEREST

The authors declare that there exist no competing interests.

## Supporting information


Figure S1–S2

Table S1

## Data Availability

The data evidence of this research is available from corresponding authors with reasonable request.
