# Supplementary material for: Novel compound heterozygous synonymous and missense variants in the MYO7A gene identified by next‐generation sequencing in a Chinese family with nonsyndromic hearing loss
Source: J Clin Lab Anal. 2022 Sep 26;36(11):e24708. doi: 10.1002/jcla.24708 (PMC9701874; doi:10.1002/jcla.24708)
Supplement: Supplementary file 1 — Figure S1–S2 [file JCLA-36-e24708-s002.docx]

**Supplementary material file 2**

Figure S1 Normal retina and optic disc were evident in the left (A) and right (B) eye of the proband by fundus ophthalmoscopy


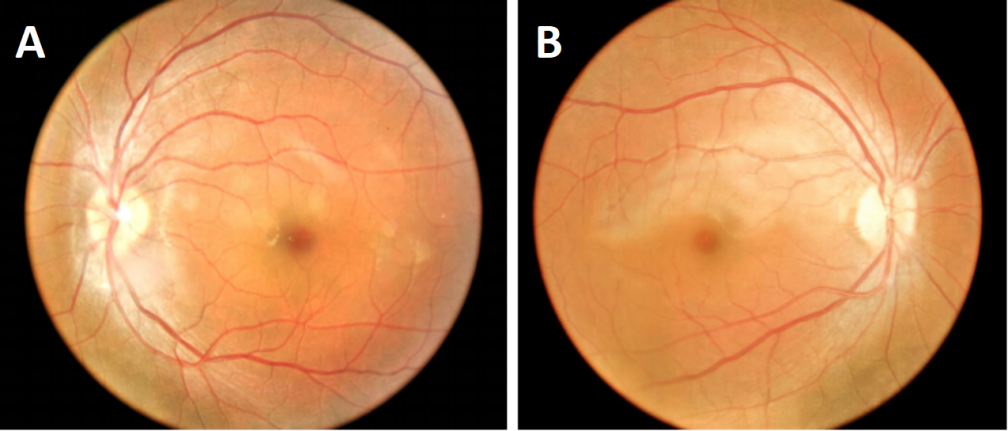


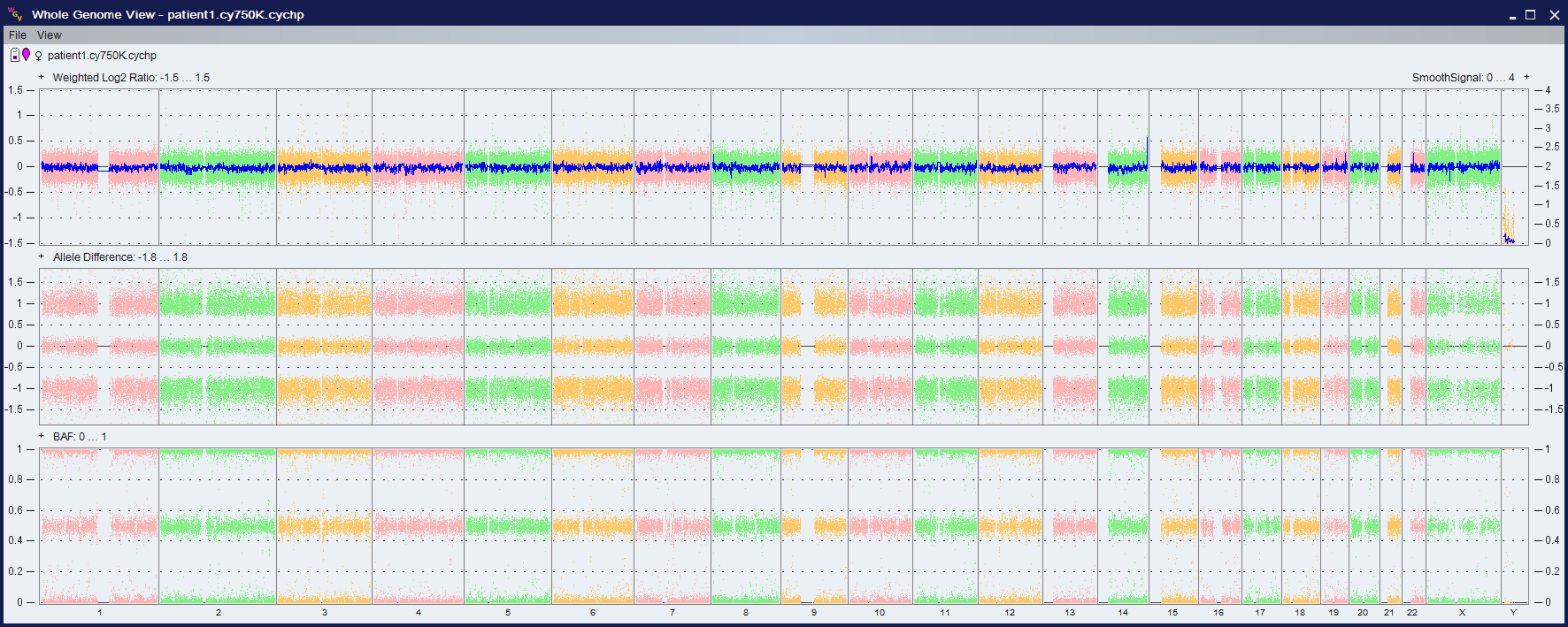
Figure S2 No potential pathogenic CNVs were detected by chromosomal microarray analysis in the proband
